# A Lifelong Battle With Crouzon Syndrome: A Detailed Case Report of Extensive Craniofacial Surgeries and Complex Psychiatric Care

**DOI:** 10.7759/cureus.91605

**Published:** 2025-09-04

**Authors:** Sai Tejaswi Gillela, Palash Jaiswal, Soojae Hollowell, Mihika Nepal, Luba Leontieva

**Affiliations:** 1 Psychiatry, Kakatiya Medical College, Warangal, IND; 2 Psychiatry, Gujarat Cancer Society Medical College, Ahmedabad, IND; 3 Psychiatry, State University of New York Upstate Medical University, Syracuse, USA

**Keywords:** borderline personality disorder (bpd), craniofacial surgeries, crouzon syndrome, post-traumatic stress disorder (ptsd), substance abuse

## Abstract

Crouzon syndrome (CS) is a rare genetic disorder characterized by the premature fusion of cranial sutures, leading to distinctive facial features and potential neurological complications. Individuals with craniofacial syndromes often face psychosocial challenges that may increase their vulnerability to mental health issues. This case report details a 55-year-old woman with CS who endured multiple complex surgeries, including brain surgery and Le Fort III advancement osteotomy, during her early years. Having experienced neglect by her biological parents and abuse within her foster care environment, these traumatic experiences likely contributed to her heightened risk for mental health issues. She was later diagnosed with post-traumatic stress disorder and borderline personality disorder and had a history of substance abuse. Despite the prevalence of such psychiatric comorbidities, the intersection between CS and mental health disorders is rarely examined in the literature. This case emphasizes the need for more research and highlights the importance of an integrated care approach to address medical and psychiatric complexities in patients with CS.

## Introduction

Crouzon syndrome (CS) is a rare but striking genetic disorder that causes craniofacial dysostosis, typically resulting from a mutation in the fibroblast growth factor receptor gene, most often FGFR2. With a global incidence of just 16.5 cases per million [1], its early clinical presentation and lifelong complications present a complex challenge for both surgical and psychosocial management.

The mutation of FGFR2 results explicitly in premature ossification of 2 or more sutures of the skull. The growth potential of the skull is restricted if the sutures fuse prematurely [2]. CS significantly impacts the development of the skull and facial bones, leading to a spectrum of craniofacial abnormalities. The physical manifestations of the syndrome can range from mild to severe and typically include wide-set eyes (hypertelorism), bulging eyeballs (proptosis), crossed eyes (strabismus), a prominent forehead, a small, beak-shaped nose, an underdeveloped jaw, and, in some cases, a cleft lip or palate. These distinct features can lead to various complications, such as vision and dental problems, hearing loss, breathing difficulties, and hydrocephalus [3]. Although intellectual disability is rare in CS, it can still occur and requires careful monitoring [4].

These complications underscore the importance of early diagnosis and multidisciplinary management. While the surgical aspects of CS are well described, the long-term psychiatric effects remain underrecognized. Our patient’s history of schizoaffective disorder, borderline personality disorder, post-traumatic stress disorder (PTSD) from severe sexual abuse, and substance use highlights the potential impact of repeated neurosurgical interventions and early trauma. This case offers a rare glimpse into the intersection of craniofacial disorders and chronic mental illness, emphasizing the need for trauma-informed, integrated care. To the best of our knowledge, no prior reports have explored such psychiatric comorbidities in a patient with CS.

## Case presentation

The patient was a 55-year-old Caucasian woman who presented with acute suicidal ideation, expressing intent to overdose on her medications. She had a 30-year history of borderline personality disorder and schizoaffective disorder, a 25-year history of PTSD related to childhood sexual abuse, and a 20-year history of polysubstance use. She expressed a belief that the staff and caregivers at her group facility would be better off without the financial burden of her care. Although she planned to overdose on her psychiatric medications, specifically lithium, clozapine, and Wellbutrin, she ultimately decided against it and informed the staff at her. Subsequently, she was brought to the Emergency Department (ED) for further evaluation. During the interview, the patient continued to express passive suicidal ideation. She described a progressive decline in self-care over the past two weeks, including not sleeping, eating, or showering, and expressed a sense of hopelessness. She stated that she stopped taking her medications for two days, feeling they were ineffective.

On a mental status examination, the patient was a 55-year-old woman who was oriented to time, place, and person. She was cooperative, did not have psychomotor agitation or psychomotor retardation. Her speech was spontaneous, with a normal rate, volume, and quantity. Her mood was reported as “Bad,” with a tearful yet stable affect that was appropriate and congruent with her stated mood. The thought process appeared linear, coherent, and goal-directed, with content revealing passive suicidal ideation. She reported auditory and visual hallucinations. She described the auditory hallucinations, which involved voices of her adoptive father and uncle, and visual hallucinations as "black figures without faces," which she felt were following her. The patient demonstrates fair insight, and she inconsistently adheres to the prescribed treatment plan. Furthermore, the patient's judgment is poor, as evidenced by her impulsive decisions, such as formulating plans for overdosing.

Background

The patient was diagnosed with CS at birth, was abandoned by her biological parents, and spent her early years in foster care. She had an extensive history of abuse while living in her adoptive home, which she continues to experience flashbacks of. Throughout her childhood, she faced significant bullying at school due to her facial features, which made it difficult for her to form friendships and left a profound emotional impact.

**Figure 1:**
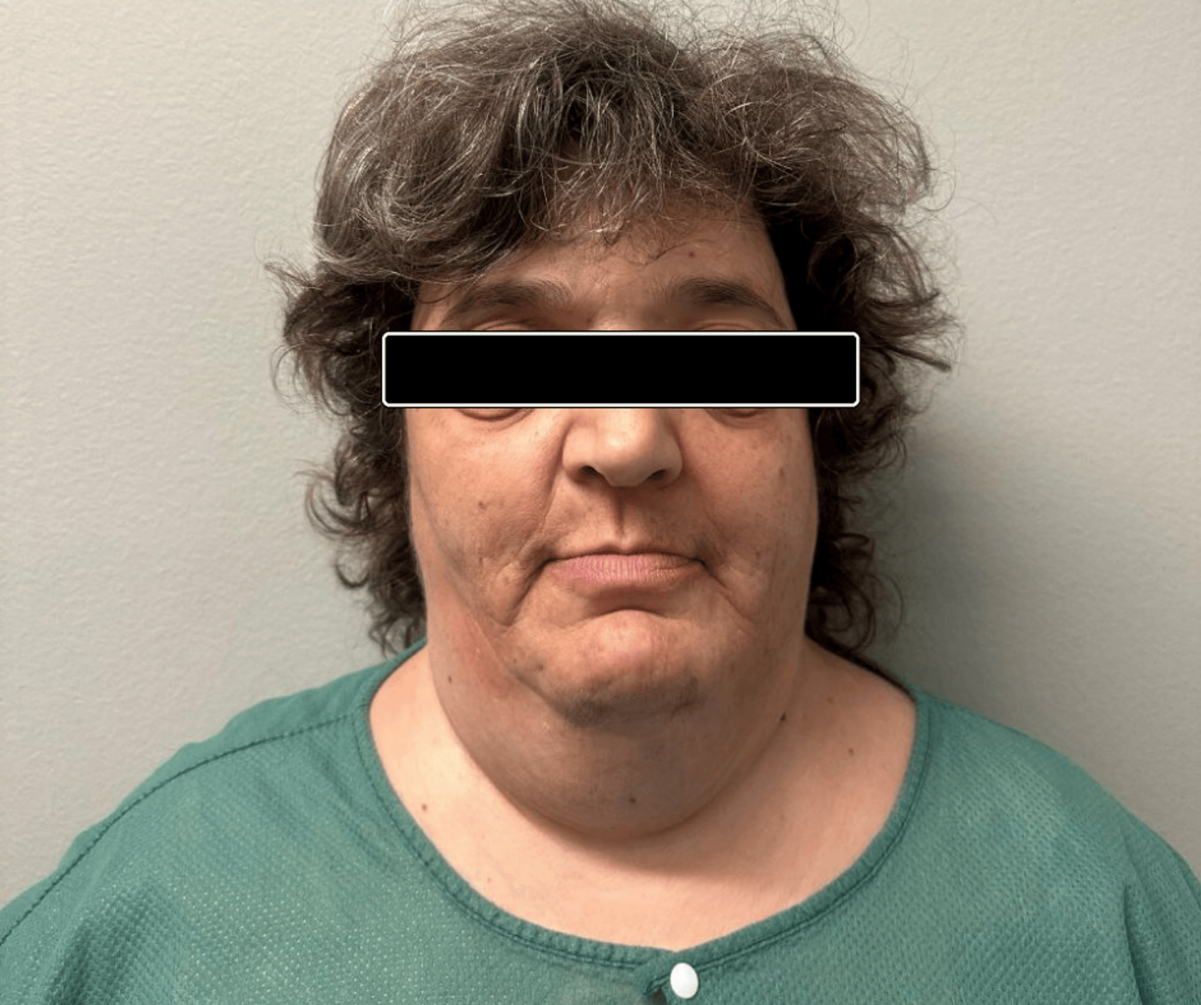
Frontal view of the patient showing characteristic craniofacial features of Crouzon syndrome. Facial features of the patient demonstrate characteristic craniofacial abnormalities consistent with Crouzon syndrome, including frontal bossing, hypertelorism, and midface retrusion. Written informed consent to include the image in a published article was obtained from the patient.

**Figure 2:**
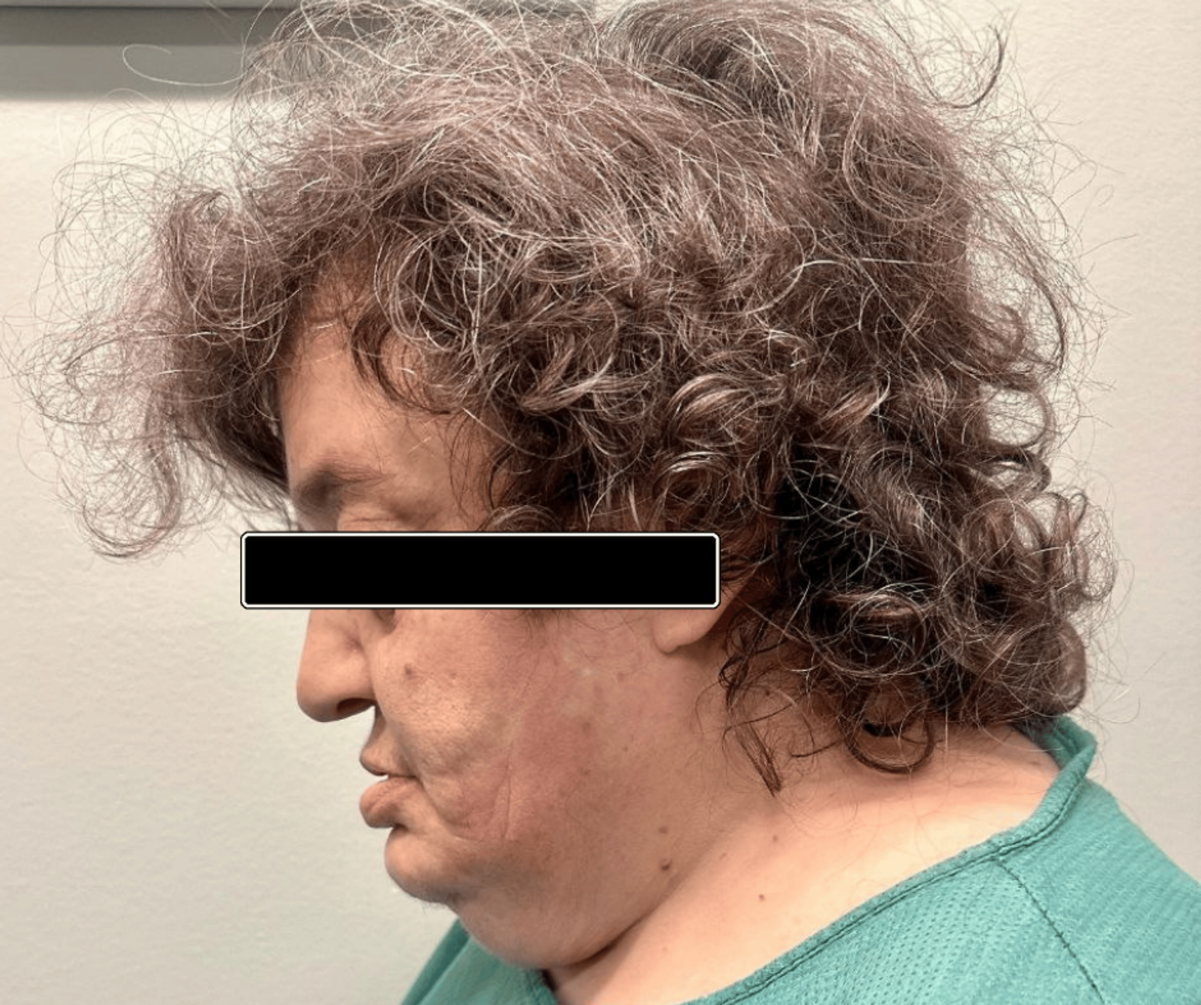
Lateral view of the Crouzon syndrome patient. Facial features demonstrate midface retrusion, prominent forehead, and craniofacial abnormalities characteristic of Crouzon Syndrome. Written informed consent to include the image in a published article was obtained from the patient.

During her childhood, she had a markedly retruded midface and nose with a class III malocclusion, because of which she suffered from breathing difficulties. During adolescence, she experienced hydrocephalus, necessitating combined craniofacial and neurosurgical interventions. The last major procedure was a forward face reconstructive surgery (Le Fort III advancement osteotomy) in 1985, when she was 17 years old. Despite undergoing surgeries, she continues to exhibit the hallmark features of CS, including a prominent forehead, hypertelorism, strabismus, beaked nose, short upper lip, hypoplastic maxilla, and relative mandibular prognathism (Figures 1, 2).

The patient has a long history of psychiatric hospitalizations beginning in her early 20s, with the first admission at age 22 for self-harming behavior. She was later hospitalized following a minor incident in which she accidentally set papers on fire at her group facility and tested positive for amphetamines. This was not considered a deliberate act of arson, and no other fire-setting episodes have been reported. More recent admissions were due to medication non-compliance, depression, and lithium toxicity. She continues to struggle with suicidal ideation, multiple suicide attempts, and the lasting effects of childhood sexual trauma.

Past Medical History

The patient's medical history is extensive and complex, encompassing multiple chronic conditions, surgeries, and significant past medical events. This unique medical history underscores the complexity of her case and the need for a comprehensive approach to her care.

Chronic conditions: Diabetes mellitus type 2 (DM 2), essential hypertension, hepatitis C, lactose intolerance, asthma, sleep apnea, gastroesophageal reflux disease (GERD), chronic rhinitis, sinusitis, morbid obesity, Barrett’s esophagus, substance abuse, migraine, and hearing loss. 

Cardiovascular conditions: Mitral valve prolapse, complete heart block, and acute biliary pancreatitis. 

Surgical history: Brain surgery, Le Fort III and Le Fort I advancement osteotomies of the midface, intermaxillary fixation, cardiac pacemaker, colon surgery, cholecystectomy, and tonsillectomy.

Family History and Social History

The patient’s family history is unknown, as she was adopted from foster care at the age of eight. She attended school until the 9th grade and later earned a General Educational Development (GED), but eventually dropped out of a business institute. She has never been employed and receives disability benefits. The patient was raised in her adoptive home, where she shared a close bond with her adoptive mother. In her twenties, she transitioned to a supervised nursing facility, where she followed a structured routine. She maintained a small circle of friends, spent her time playing board games, and generally spent her days engaged in meals, games, and rest.

Substance use history: The patient began using alcohol at the age of 8, influenced by her adoptive family. She reports drinking "as a crutch to relieve the pain." During her teenage years, she started using street drugs, primarily abusing marijuana. Her substance use escalated to include cocaine. She reports that she last drank alcohol in 2006 and last used marijuana in 1991.

Course during inpatient treatment

Upon admission to the inpatient psychiatry unit, the patient reported feeling depressed and paranoid about those around her, becoming increasingly isolated, and expressing concerns that her medications were no longer effective. A review of her medication regimen revealed low levels of lithium (0.42, therapeutic range: 0.6-1.2 mEq/L) and clozapine (169), prompting an increase in dosages: lithium was raised to 900 mg, clozapine to 300 mg, and Wellbutrin to 300 mg to address worsening depressive symptoms. Her other medications, atorvastatin 80 mg PO once daily, Bydureon BCise 2 MG/0.85ML extended-release injection (2 mg, subcutaneous, every 7 days), dexlansoprazole 60 mg, PO once daily, metformin 500 MG 24 hr tablet, topiramate 100 MG tablet PO twice daily, were continued at the same doses.

The patient was assessed using the Positive and Negative Syndrome Scale (PANSS; Kay et al., 1987), specifically focusing on item N5 (Difficulty in Abstract Thinking), along with the Similarities and Proverbs subtests [5]. We applied the scoring guide Leontieva et al. 2019 [6]. The patient scored seven on Similarities (Appendix A; 4 is the ideal score, 16 is the worst score) and 13.25 on Proverbs (Appendix B; 4 is the perfect score, 16 is the worst score). These scores indicate significant difficulties in abstract thinking and prominent concreteness. We also administered the Pictogram Test (PT; Leontieva et al., 2008) to the patient [7]. This is the test of associative memory that taps into associative thinking. The patient has 59% recall; her associations were very concrete and repetitive. Together, the PANSS-N5 and the PT corroborate significant difficulty in abstract thinking and concrete thinking, both seen in patients with organicity (developmental delay, brain surgeries).

The patient adhered to the treatment plan throughout the course and showed significant improvement. She reported a better mood, actively participated in various therapeutic sessions like group psychotherapy and dialectical behavior therapy (DBT) sessions, each lasting approximately 60 minutes and attended twice weekly, and engaged in self-reflection. These interventions contributed to a decrease in auditory hallucinations, and at the time of discharge, she denied any auditory or visual hallucinations. Her sleep also improved, though she continued to experience occasional nightmares and used as-needed medications when feeling restless. At discharge, the patient was cooperative, calm, and future-oriented. She denied any significant symptoms of depression, mania, anxiety, or psychosis, including changes in sleep, mood, anhedonia, fatigue, and active/passive suicidal or homicidal ideations.

The patient was discharged from the acute inpatient unit, with an appointment with her outpatient providers scheduled.

## Discussion

This case offers valuable insights into the intricate relationship between physical and mental health in individuals with CS. The patient’s psychological development was shaped by multiple factors, including the challenges of living with CS, early parental abandonment, and childhood trauma. Her early exposure to numerous invasive surgeries further compounded her emotional vulnerability, contributing to the development of psychiatric conditions. These experiences disrupted her ability to form healthy attachments, impacted her self-identity, and impaired her coping strategies, highlighting the profound impact of early life adversity on long-term mental health in patients with CS.

Genetic factors

Diagnosed with CS at birth, she underwent a series of intricate surgeries during her early childhood, each contributing to a profoundly distressing and challenging experience. Premature synostosis of the cranial sutures, a hallmark of CS, often begins in utero and manifests at birth, involving the coronal, sagittal, and sometimes lambdoid sutures. The premature fusion of these sutures restricts growth perpendicular to the fused suture, resulting in abnormal bone development, particularly in the skull and face. As seen in our patient, compensatory growth at the remaining open sutures leads to significant facial deformities. This necessitated early surgical intervention to allow for proper brain growth and skull expansion [8]. 

Her last surgery was facial reconstructive surgery at the age of 17 to correct midfacial hypoplasia, shallow orbits, and upper airway obstruction caused by premature skull base fusion. She initially suffered from breathing problems, which were resolved after her surgeries, which is consistent with the literature that highlights how upper airway obstruction is a significant concern in CS [9]. This staged surgical approach aligns with current best practices in treating craniofacial dysostosis syndromes, where early release of synostotic sutures is essential for cranial volume expansion, and subsequent surgeries are performed as the patient grows to optimize functional and cosmetic outcomes [10]. It is one of the few syndromes where cosmetic surgery results may be very effective [11].

Despite the general observation that mental capacity in CS patients is usually within the normal range, a few experience some mental delay, possibly attributable to the increased intracranial pressure during their development [12]. Our patient’s IQ was reported to be 64. She was approximately four years behind in achieving developmental milestones.

Early life stress

During her formative years, she encountered substantial social adversity due to her facial deformities, which resulted in neglect by her biological parents at birth and subsequent placement in foster care. After being adopted, she endured abuse within her adoptive family, leaving a profound emotional impact. Her upbringing was marked by limited social connections, with her emotional world primarily centered around a close yet fragile bond with her adoptive mother. This dynamic fostered a pattern of dependency and heightened emotional vulnerability throughout her development. The patient’s life underwent a profound shift following the death of her adoptive mother, which precipitated a surge in suicidal ideation and led to multiple psychiatric hospitalizations. 

Previous studies indicate that early life stress (ELS) affects child development in behavioral, emotional, social, physical, and cognitive areas. Traumatic events experienced during development may damage neurobiological and neuroendocrine aspects, which remain for the rest of one’s life [13]. Research shows that neglect during childhood, especially for those with physical differences, is strongly associated with severe psychiatric outcomes, including psychosis, depression, and substance use disorders in adulthood [14]. Emotional neglect can result in complex PTSD and hinders the development of coping mechanisms, leading to higher vulnerability to stress and psychiatric symptoms [15,16]. 

Severe bullying and social isolation: She experienced severe bullying at school and struggled with a lack of friends. Research indicates that individuals with craniofacial conditions, such as CS, often face significant challenges to their social and psychological well-being. Patients often face social stigmatization, resulting in low self-esteem and poor body image. They experience higher levels of psychological distress and lower quality of life (QoL) compared to their peers. This distress is further compounded by bullying and social isolation, contributing to long-term mental health challenges [17]. These psychosocial stressors likely compounded her overall health challenges.

Our patient’s treatment journey underscores the complexity of managing CS, where multiple staged surgeries are necessary for functional improvement and significant cosmetic enhancement. The multidisciplinary approach involving neurosurgery, plastic surgery, and psychosocial support is crucial for achieving the best possible outcomes for these patients. Additionally, genetic counseling and information are essential for parents with an infant diagnosed with CS. Genetic testing of the initial case can be considered for patients with atypical presentations or those seeking a prenatal diagnosis. Providing psychosocial support and assisting families in accepting their child's condition is essential for empowering them to support their child's development and potential.

Given this perspective, symptomatic treatment, along with the use of hearing aids, phonotherapy, psychoeducation, family counseling, genetic advice, speech therapy, lip reading, as well as attending a unique or high-quality school, all contribute to enhancing the quality of life for individuals with this syndrome.

## Conclusions

Our patient’s journey highlights the importance of a long-term, multidisciplinary approach to managing Crouzon syndrome. This case illustrates the need for early intervention, continuity of psychiatric care, and a supportive environment that addresses both physical and mental health. Recognizing the psychosocial challenges faced by patients with craniofacial syndromes is essential to ensuring holistic care. Patients with facial deformities often experience hidden vulnerabilities that profoundly affect their well-being, and addressing these concerns early in life by integrating medical and psychiatric care is crucial for their overall emotional and psychological development.

This case further underscores the need for greater recognition of the psychiatric sequelae that may accompany rare congenital disorders like Crouzon syndrome, particularly in individuals with histories of trauma and prolonged institutionalization. Future efforts should focus on developing integrated care models that bring together psychiatry, genetics, craniofacial surgery, and rehabilitative services. Longitudinal research is warranted to explore long-term psychiatric outcomes in patients with craniofacial syndromes, as well as the efficacy of trauma-informed, multidisciplinary interventions. Early identification of psychosocial vulnerabilities and consistent mental health support may improve both functional outcomes and quality of life in this population.

## Figures and Tables

**Figure 3:**
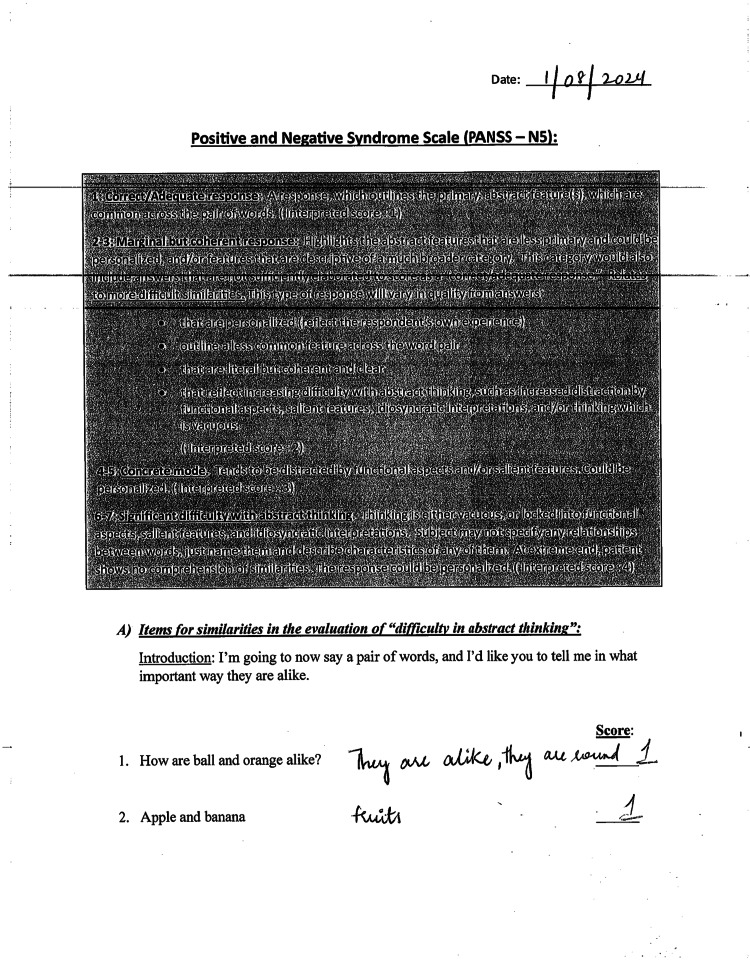
Similarities (Page 1)

**Figure 4:**
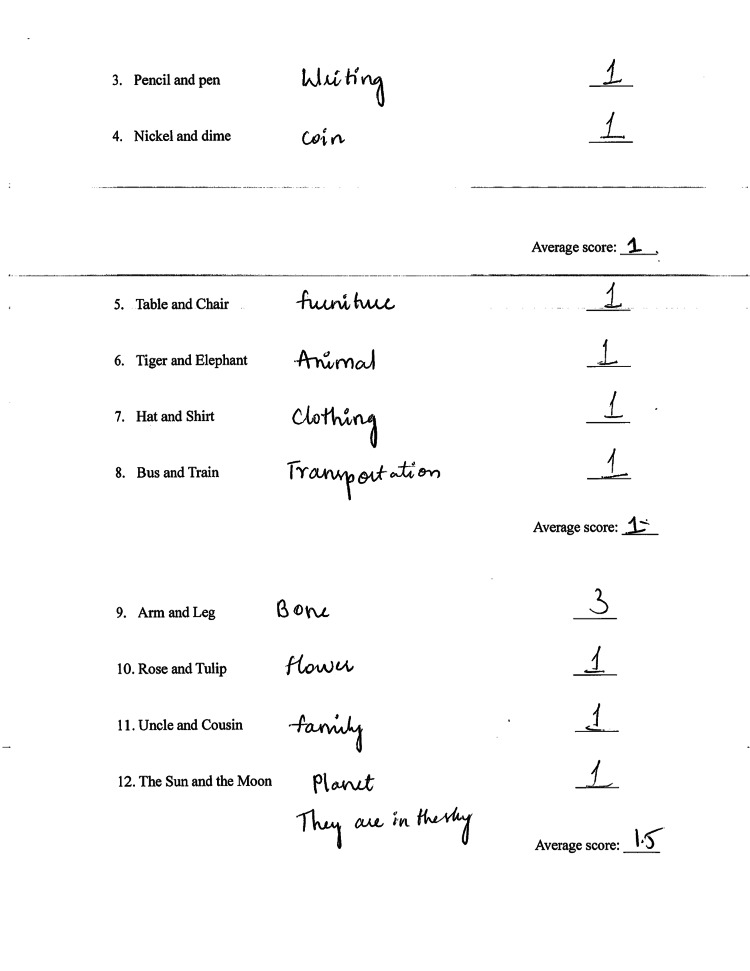
Similarities (Page 2)

**Figure 5:**
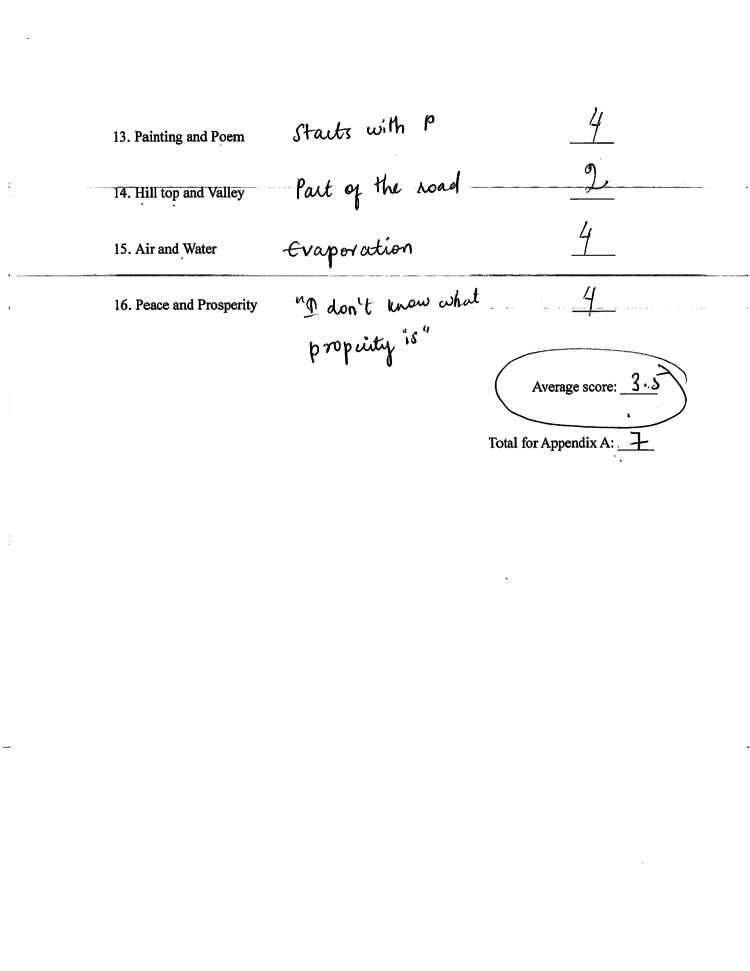
Similarities (Page 3)

**Figure 6:**
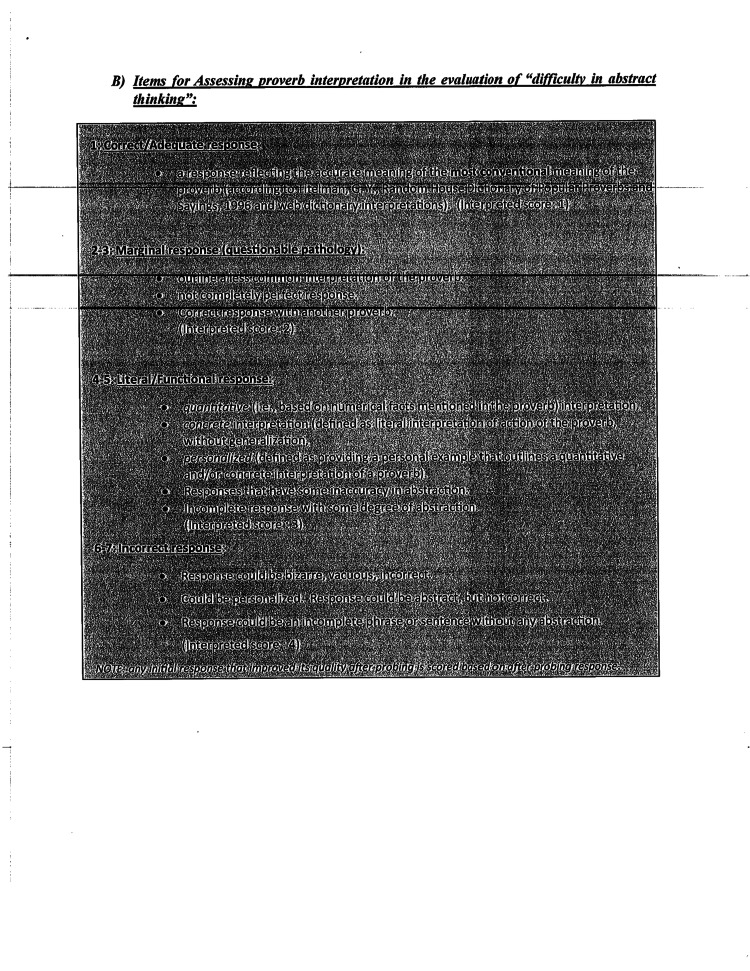
Proverbs (Page 1)

**Figure 7:**
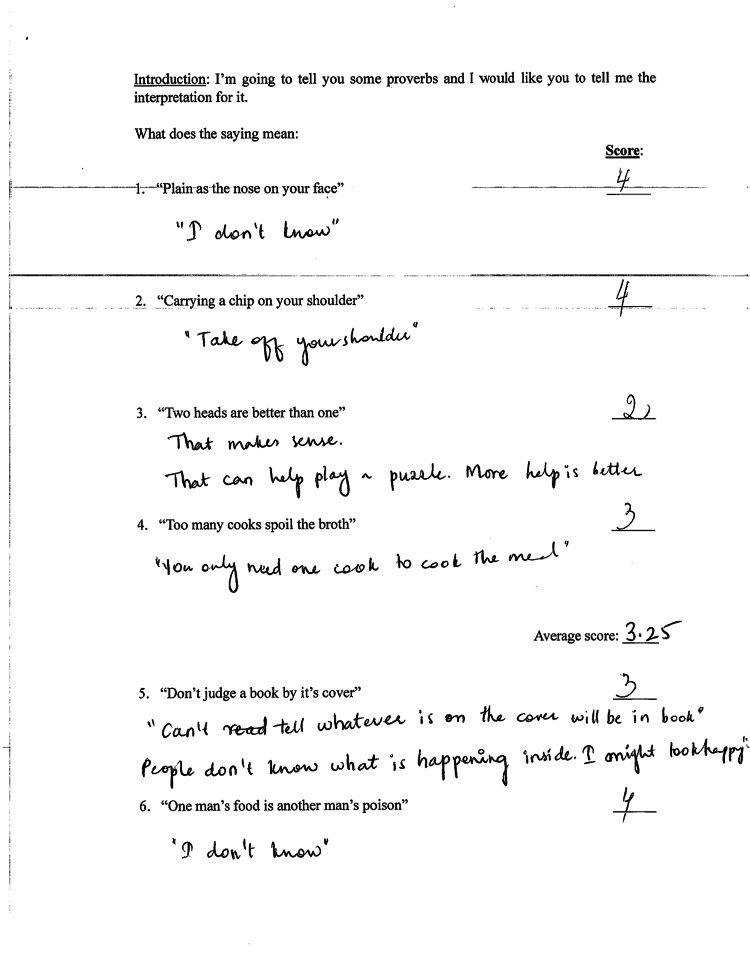
Proverbs (Page 2)

**Figure 8:**
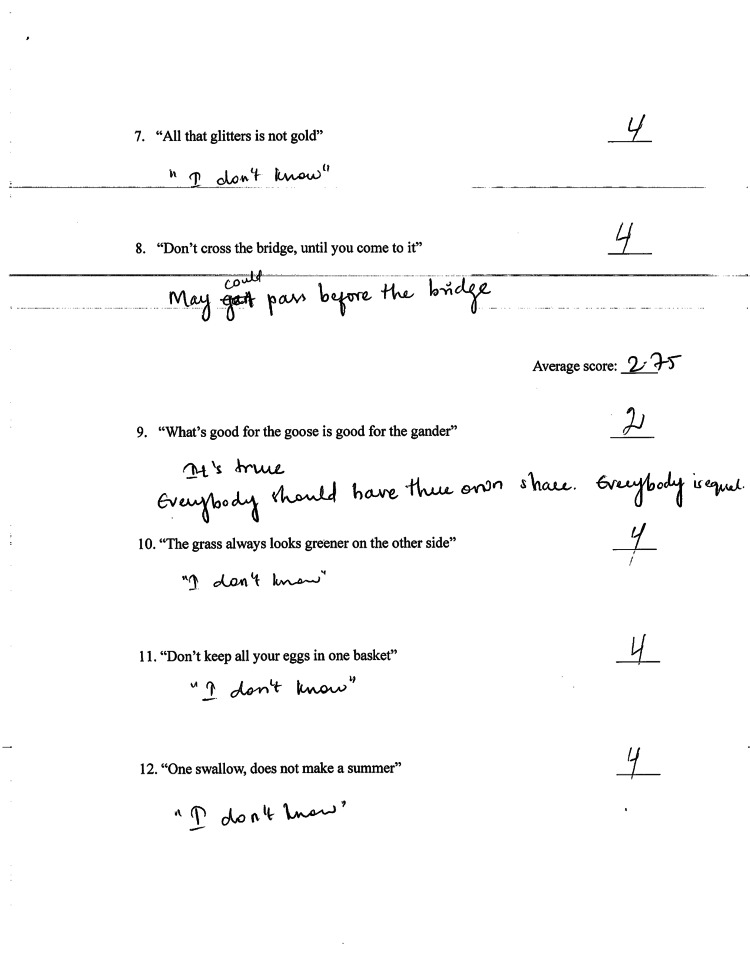
Proverbs (Page 3)

**Figure 9:**
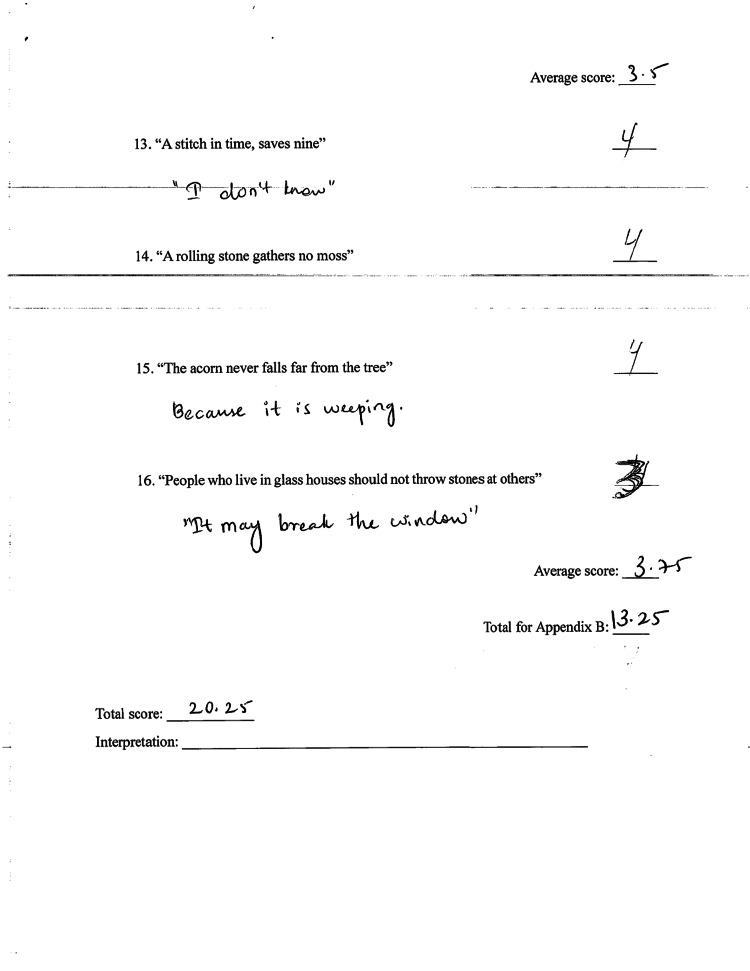
Proverbs (Page 4)

## Appendices

Appendix A

Appendix B
